# In Situ Observation of Cellular Structure Changes in and Chain Segregations of *Anabaena* sp. PCC 7120 on TiO_2_ Films under a Photocatalytic Device

**DOI:** 10.3390/molecules28207200

**Published:** 2023-10-20

**Authors:** Xiaoxin Wang, Jingtao Zhang, Qi Li, Ran Jia, Mei Qiao, Wanling Cui

**Affiliations:** 1 College of Physics and Electronics, Dezhou University, Dezhou 253000, China; jiaran0518@163.com (R.J.); qiaomeirr@sina.com (M.Q.); 2 Shandong Provincial Key Laboratory of Biophysics, Dezhou University, Dezhou 253000, China; wanlingcui@163.com; 3 Shenyang National Laboratory for Materials Science, Institute of Metal Research, Chinese Academy of Sciences, Shenyang 110016, China; jtzhang@zzuli.edu.cn (J.Z.); qiliuiuc@swjtu.edu.cn (Q.L.); 4 College of Tobacco Science and Engineering, Zhengzhou University of Light Industry, Zhengzhou 450002, China; 5 Key Laboratory of Advanced Technologies of Materials (Ministry of Education), School of Materials Science and Engineering, Southwest Jiaotong University, Chengdu 610031, China

**Keywords:** in situ optical observation, chain fracture mechanism, photocatalytic inactivation

## Abstract

Cyanobacteria outbreaks are serious water pollution events, causing water crises around the world. Photocatalytic disinfection, as an effective approach, has been widely used to inhibit blue algae growth. In this study, a tiny reaction room containing a TiO_2_ film was designed to fulfill in situ optical observation of the destruction process of a one-dimensional multicellular microorganism, *Anabaena* sp. PCC 7120, which is also a typical bacterial strain causing water blooms. It was found that the fragment number increased exponentially with the activation time. The fracture mechanics of the algae chains were hypothesized to be the combining functions of increased local tensile stress originated from the cell contracting as well as the oxidative attacks coming from reactive oxygen species (ROSs). It was assumed that the oxidative species were the root cause of cellular structure changes in and chain fractures of *Anabaena* sp. PCC 7120 in the photocatalytic inactivation activity.

## 1. Introduction

Cyanobacteria (blue algae) blooms have caused huge health threats and economic losses in aspects including aquatic food production, drinking water supplies, recreation, and tourism worldwide [1,2]. Meanwhile, the other threat brought by cyanobacteria blooms is cyanotoxins, which are released when cyanobacteria die or their cells break. Therefore, researchers focused on developing methods that could remove the cyanobacterial cells and degrade the cyanotoxins simultaneously to ensure water safety [3]. Based on traditional oxidative methods including chlorination [4] and ozone [5], advanced oxidation processes have been attempted for the removal of cyanobacteria and the degradation of cyanotoxins in a more effective, inexpensive, and eco-friendly way. For instance, ozone micro-bombs [1] and CaO_2_-functionalized alginate beads [6] were designed to eliminate the pollution caused by cyanobacteria blooms.

Photocatalytic disinfection was proven to be another potential route to solving blue algae blooms, thanks to the fundamental work of Matsunaga et al. in 1985 [7]. They found that photocatalytic disinfection could effectively disinfect the whole spectrum of microorganisms, including bacteria [8], viruses [9], and fungi [10]. For the treatment of blue algae and its related environmental problems, TiO_2_-based materials have been demonstrated to possess high photocatalytic inhibition activity for blue algae growth, and could photocatalytically degrade toxic cyanotoxins [11,12,13,14]. Immobilizing TiO_2_-based photocatalysts on appropriate substrates, such as fibers [15], foams [16], and membranes [17], makes photocatalysis a more promising approach to the treatment of cyanobacteria-contaminated water bodies. To better understand the photocatalytic disinfection of blue algae, the in situ observation of changes in blue algae during the photocatalytic treatment process is essential, which could reveal its disinfection mechanism and may provide interesting findings in biophysics.

However, no such report is available in the literature, which may be attributed to the experimental difficulty of the in situ observation of living creatures on such a small scale. In this work, we report, for the first time, the in situ observation of cellular/chain structure changes during the photocatalytic treatment of *Anabaena* sp. PCC 7120, which is a filamentous, heterocyst-forming cyanobacterium, one of the main bacterial strains causing water blooms and excreting cyanotoxins [18]. And we tried to analyze the photocatalytic fragmentation mechanism of blue algae chains through in situ observation.

## 2. Results and Discussion

**Figure 1:**
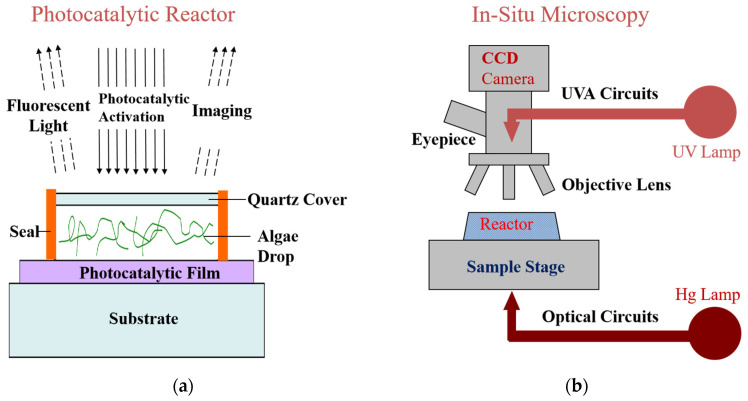
Schematic diagrams of (**a**) the micro-reaction chamber (section view) and (**b**) the optical circuits in the biological microscope.

To realize real-time, in situ observation, a micro-reaction chamber (see Figure 1a) was constructed from a UV-activated photocatalytic TiO_2_ thin film spun-coated on a glass slide from a sol–gel precursor solution (the XRD pattern and SEM image of the TiO_2_ thin film can be seen in Figure S1) [19]. After the *Anabaena* sp. PCC 7120 solution was dropped onto the TiO_2_ thin film, a quartz cover slip was put on the blue algae solution drop, and the *Anabaena* sp. PCC 7120 solution was sealed by applying a polymer adhesive along the cover slip and the TiO_2_ thin film to avoid solution loss and evaporation during the experiment. As demonstrated in Figure 1b, the micro-reaction chamber was placed on the object stage of a biological fluorescence microscope (Nikon 80i-PH-FL). The optical circuits not only provided the UV light source to activate the semiconductor TiO_2_ thin film, but also carried the optical signal from the specimen for imaging. Therefore, this technique allowed the photocatalytic reaction and the in situ microscopy observation to be made at the same time. With the help of a DAPI filter block, UVA light illumination with 340 to 380 nm was obtained to realize TiO_2_ excitation (see Figure S2). It was not necessary to add oxygen to the reaction room due to the fact that cyanobacterium is an oxygenic photosynthesis bacterium.

**Figure 2:**
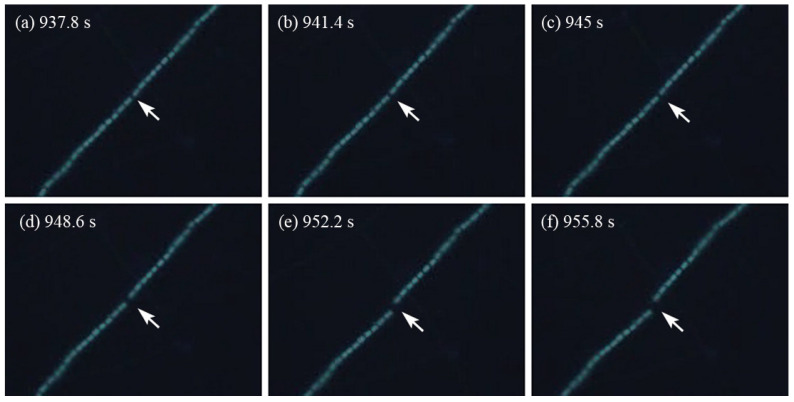
The video screenshots of chain rupture process at different photocatalysis times (The arrows indicated where the chain rupture occurred.

Figure 2 presents a series of images of an *Anabaena* sp. PCC 7120 chain exposed to the photocatalytic stimulus for various time intervals. Prior to the application of the photocatalytic stimulus, the *Anabaena* sp. PCC 7120 chain consisted of a large number of individual cells connected end-to-end. Most of the chains were long, continuous, and floating in the liquid medium with little movement. Within each chain, the distance between the adjacent cells was relatively constant. After the photocatalytic stimulus was applied for an extended period of time, the chain appeared permanently deformed and ruptured into multiple fragments. Figure 2a shows the emergence of a gap between two individual cells at the location indicated by the arrow. As the photocatalysis time continued, the gap became larger, as seen in Figure 2b–d, and eventually, the chain broke into separate pieces (Figure 2e,f). The chain rupture apparently occurred along the septum of the cyanobacteria. After the chain rupture, the cells in the broken pieces were still active, continuing to vibrate at a fast speed, as the photocatalytic stimulus remained in place. The dynamic process of the chain rupture was captured by the video camera mounted on the microscope and shown in Video S1.

**Figure 3:**
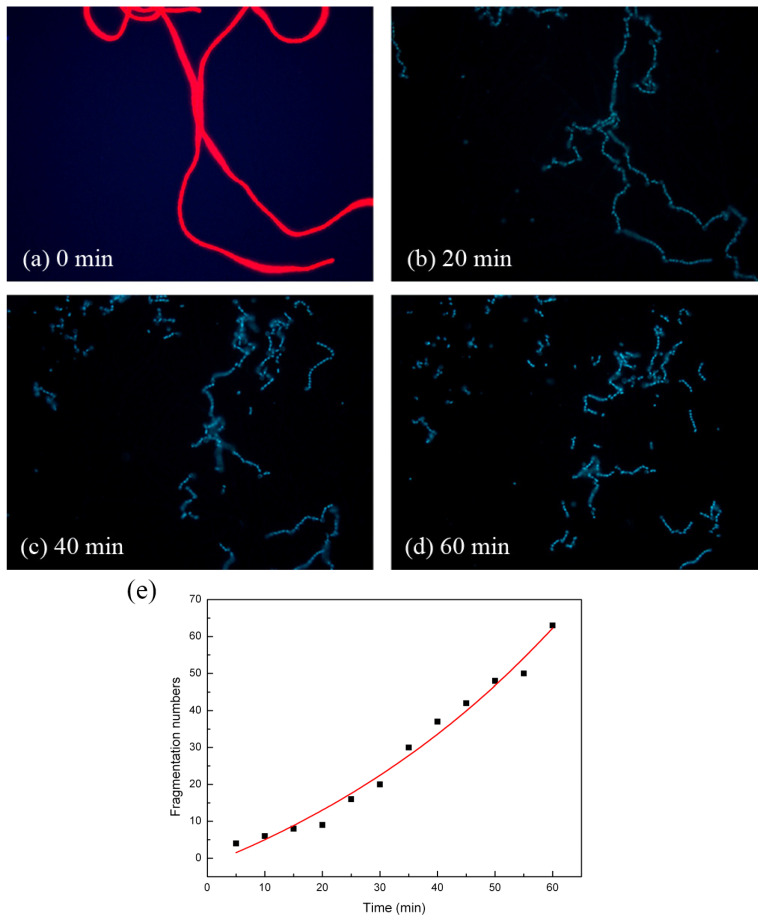
Fluorescence images of *Anabaena* sp. PCC 7120 treated with UVA on TiO_2_ film at (**a**) 0 min, (**b**) 20 min, (**c**) 40 min, (**d**) and 60 min at a magnification of 400 times. (**e**) The relationship between chain/fragment numbers and the photocatalysis time, t.

For the long *Anabaena* sp. PCC 7120 chains, the rupture occurred at multiple locations, though not necessarily at the same time, resulting in multiple fragments. As shown in Figure 3a, the field of view initially enclosed two long continuous cyanobacterial chains. At the beginning of the photocatalytic activation, the chains were deformed to assume different configurations (Figure 3b). After extended periods of photocatalytic treatment, the long, continuous chains broke into short fragments (Figure 3c,d). The longer the photocatalysis time was, the shorter the fragments were. Figure 3e demonstrates the chain/fragment numbers observed at different photocatalysis time intervals in one representative observation out of eight observations. It clearly demonstrates that the chain/fragment numbers increased generally with the increase in photocatalysis time, and the relationship between the fragment number, *N*, and the photocatalysis time, *t*, could be fit into an exponential function:

(1) $$N=\alpha e^{\frac{t}{\beta }}+\gamma$$

where fitting constants *α* = 37.62, *β* = 60.37, and *γ* = 39.37. Data of the other seven observations can be found in Figure S3 in the Supplemental Materials, all of which followed similar exponential functions.

**Figure 4:**
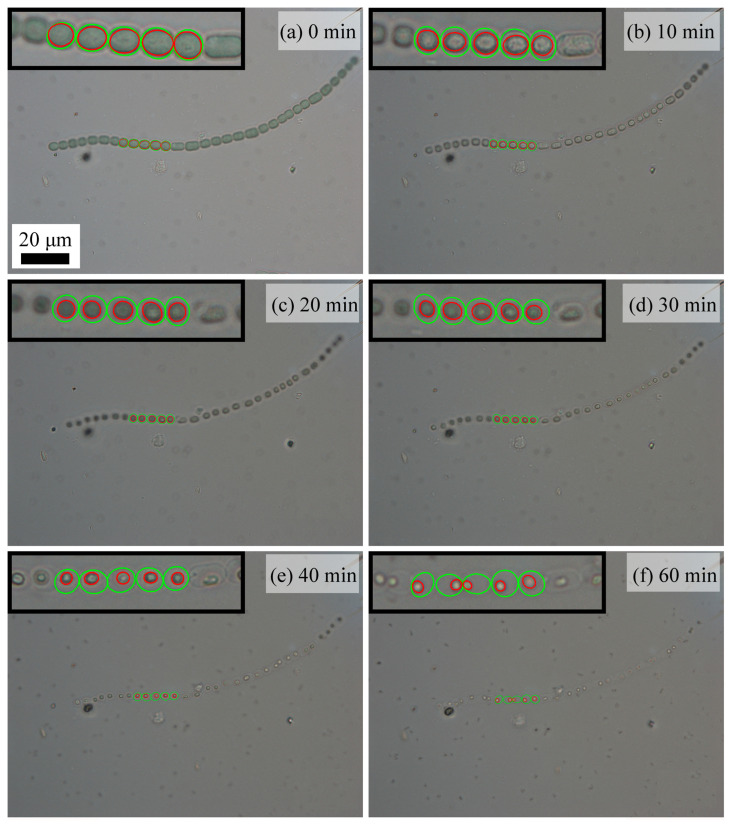
Optical observations of morphology changes in *Anabaena* sp. PCC 7120 treated with UVA on TiO_2_ film at (**a**) 0 min, (**b**) 10 min, (**c**) 20 min, (**d**) 30 min, (**e**) 40 min, (**f**) and 60 min at a magnification of 1000 times (The green circles indicated the cells chosen for the average apparent cellular area decreasing calculation, and the red circles indicated the protoplasm chosen for the average apparent protoplasm area decreasing calculation).

To relate the chain rupture with individual cell behavior, the morphology of individual cells was examined as a function of the photocatalysis time. Figure 4a–f demonstrates the morphology changes in one *Anabaena* sp. PCC 7120 chain during 1 h of photocatalytic treatment. Five connected cells (cells numbered 9 to 13 from the left end of this chain) were used to examine the average cell/protoplasm area changes. Green circles were used to mark the cells, while red circles were used to mark the protoplasm in the cells. When there was no photocatalytic stimulus (Figure 4a), the cells in this chain were plump, and their protoplasm was uniformly distributed in the cells. After 10 min of photocatalytic treatment (Figure 4b), however, the cells began to shrink, and their protoplasm also shrank and became partly detached from the cell wall. With the increase in photocatalysis time (Figure 4b–f), both shrinkage in the cell/protoplasm area and the cell wall/protoplasm detachment were largely enhanced.

**Figure 5:**
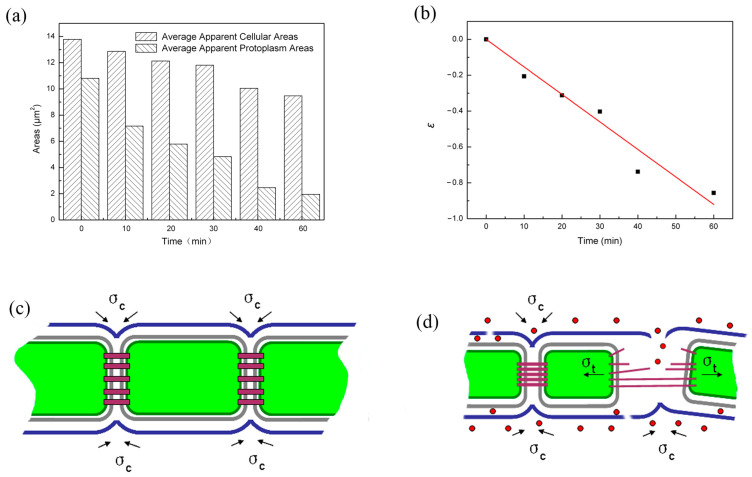
(**a**) The average apparent cellular areas and protoplasm areas at different time intervals during the photocatalytic treatment process. (**b**) The relationship between the cellular strain and the photocatalytic treatment time, t. (**c**) Schematic diagram of the chain structure. (**d**) Schematic diagram of the chain fracture mechanism during the photocatalytic treatment (Note: red dots represent reactive oxygen species, and the arrows indicate the stress directions).

For a chain link made of a series of individual rings, the entire chain simply goes through a translational movement if each ring shrinks freely. However, if the chain is constrained to two fixed points, the segment in between will be placed under tension. Real-time observations of the chain motion showed that as the long cellular chains took convoluted configurations in the solution (Figure 3a), points of the chain entanglement or locations with a very small radius (3–5 cell dimensions) acted as “fixed hinges” around which the chains rotated or twisted and between which individual cells vibrated. Between the hinges, the cellular chain may be viewed as a segment in the chain link analogy. At the same shrinkage strain, the greater the number of rings in the segment, the higher the tensile stress will be in the chain. When the tensile stress reaches the strength of the chain link, the chain will rupture. Based on this analogy of chain link fracture, the deformation in each cell was analyzed, and a model for the chain rupture is illustrated in Figure 5. Figure 5a demonstrates the average apparent cellular area changes in both the cells and their protoplasm calculated from cells numbered 9 to 13 during 1 h of photocatalytic treatment. The average apparent cellular area decreased from ~13.8 µm^2^ to ~9.5 µm^2^ after 1 h of photocatalytic treatment, representing a 31% decrease, while the average apparent protoplasm area had an even larger percentage decrease, from ~10.8 µm^2^ to ~1.95 µm^2^, after 1 h of photocatalytic treatment, representing an 82% decrease. The change in the apparent cellular area can be converted into a radial strain, *ε*:

(2) $$\varepsilon =\ln(1+\frac{\Delta r}{r_{0}})$$

where *r*_0_ is the initial radius, and Δ*r* is the change in the radius of the individual cell calculated from the apparent cell area by assuming a circular geometry. Using the protoplasm data, the cellular strain, *ε*, showed a linear relationship with the photocatalysis time, *t*, as shown in Figure 5b, which could be fitted into the following equation:

(3) ε=−λt

where the fitting constant *λ* = 0.015. The contraction of the cell induces tensile stress, whose magnitude depends on the stress strain or constitutive relationship of the cell membrane. Fung showed that the constitutive equation of the biological materials may be expressed by the following mathematical expression [20,21]:

(4) $$\sigma =\left(C+B\right)e^{A(\varepsilon -\varepsilon *)}-B$$

where *A*, *B*, and *C* are material constants, *ε* is the stretch strain, and ε* is a characteristic strain. Converting the shrinkage strain to the stretch strain and substituting Equation (3) into Equation (4), we arrived at the following expression for the stress in each cell:

(5) $$\sigma =\left(C+B\right)e^{A(\lambda t-\varepsilon *)}-B$$

For a fixed chain segment made of *m* cells, the total stress buildup, *T*, would be:

*T* = *mσ* (6)

As with the fracture of other materials, supposing that when the total stress, *T*, reaches a critical value, *T_c_*, the rupture strength of the septum material, the chain ruptures, we then reach the following rupture condition:

(7) $$m[\left(C+B\right)e^{A(\lambda t-\varepsilon *)}-B]=T_{c}$$

Since the number of cells in a chain fragment is directly proportional to the inverse of the number of fragments, *m = k/N*, with *k* being the proportional constant, Equation (7) may be written into the following expression:

(8) $$N=\Gamma e^{\eta t}+H$$

where $\Gamma =\frac{k\left(C+B\right)}{T_{c}}e^{-A\varepsilon *}$, *η* = *Aλ,* $H=-\frac{kB}{T_{c}}$.

It can be easily seen that Equation (8) has the same form as Equation (1), which demonstrates that our theoretical analysis was in accordance with the observed experimental result.

From the real-time, in situ microscopic observations and the chain fracture mechanical analysis, a chain fracture mechanism was proposed for the *Anabaena* sp. PCC 7120 chain under photocatalytic treatment. Figure 5c shows the schematic illustration of a simplified *Anabaena* sp. PCC 7120 chain structure, and the inside cell components are omitted. The cells in a *Anabaena* sp. PCC 7120 chain are surrounded by a plasma membrane and a peptidoglycan layer, while the whole chain structure is covered by an outer membrane [22]. In the center of the intercellular septa, the microplasmodesmata exist as the cell–cell connections, serving as the transport structure for metabolites and regulators between cells of a cyanobacterial filament [23]. The outer membrane continuously surrounds the entire chain structure and does not enter the septum between two vegetative cells [22]. To connect the two adjacent cells and maintain the chain structure, the outer membrane maintains cohesive stress (*σ*_c_) on the cells. Figure 5d shows how the chain ruptured from photocatalytic treatment. Former studies have provided strong proof of the efficient reactive oxygen species (ROSs) generation of anatase TiO_2_ film [24,25,26]. Under the attack of ROSs, the outer membrane was damaged. ROSs could go through the damaged outer membrane and cause the observed shrinkage of protoplasm. When protoplasms in two adjacent cells shrank towards the opposite directions, local tensile stress (*σ*_t_) developed in their connecting parts. With the increase in the photocatalytic treatment time, the local tensile stress could increase with their shrinkage increase, while the outer membrane around the connecting parts was continuously weakened from damage by the attack of the ROSs. When the local tensile stress finally could not be balanced by the cohesive stress from the weakened outer membrane, the algae chain ruptured at these weakened connection parts.

Moreover, control experiments were involved to eliminate the effects of UVA illumination, temperature increase, and fluid loss. When there was no TiO_2_ thin film, however, no visible difference could be observed on the *Anabaena* sp. PCC 7120 chain structure before and after UVA irradiation for 60 min, as demonstrated in Figure S4c and Figure S4d, respectively. Thus, this observation demonstrated that UVA irradiation itself could not cause huge damage to *Anabaena* sp. PCC 7120. The huge structure change demonstrated in Figure 2 and Figure 3 could only be attributed to the photocatalytic treatment with a TiO_2_ thin film in the micro-reaction chamber. Although UV irradiation had been demonstrated as a treatment option for cyanotoxins [27], cyanobacterium has evolved various strategies of UV tolerance during their long-time evolution as the oldest oxygenic inhabitants on the planet [28]. A similar observation was reported on *Anabaena* sp. [29]; no significant effect on lipid peroxidation, chlorophyll bleaching, or its survival was observed under UVA irradiation, and only UVB irradiation (the wavelength range of 280 nm to 315 nm) could cause huge damage on the algae ultrastructure and related metabolic functions.

## 3. Conclusions

Our real-time, in situ microscopy observations of the destruction process of a one-dimensional multicellular microorganism showed that multicellular organism destruction could occur at both the individual cell and the system levels when the multicellular system is attacked by ROSs generated from photocatalysis. In the case of *Anabaena* sp. PCC 7120, the cells contracted at the individual cell level. However, at the system level, the entire cellular chain suffered from increasing levels of tension as the oxidative attack continued until the chain ruptured into shorter and shorter fragments. Analysis of the mechanics of the chain rupture process provided a theoretical explanation for the experimental finding that the fragment number increased exponentially with the activation time.

## Data Availability

The data presented in this study are available upon request from the authors.

## Supplementary Materials

The following supporting information can be downloaded at https://www.mdpi.com/article/10.3390/molecules28207200/s1, where media and conditions for cell cultivation, preparation, and the characterization of TiO_2_ film can be found. Figure S1: (a) XRD pattern and (b) SEM image of anatase TiO_2_ thin film; Figure S2: (a) Schematic illustration of the filter block DAPI (Ex 340–380 nm/DM 400 nm/BA 435–485 nm) and (b) the DAPI tag along with the light source; Figure S3: The relationship between chain/fragment numbers and the photocatalysis time, t, in other 7 observations; Figure S4: OM observations of *Anabaena* sp. PCC 7120 (a) treated with UVA on TiO_2_ film for 0 min, (b) treated with UVA on TiO_2_ film for 60 min, (c) treated with UVA for 0 min, and (d) treated with UVA for 60 min at a magnification of 200 times; Video S1: The video of chain rupture process at different photocatalysis times. References [19,30,31] are cited in the Supplementary Materials.

## Sample Availability

Samples of the TiO_2_ film are available from the authors.
