# Supplementary material for: In Situ Observation of Cellular Structure Changes in and Chain Segregations of Anabaena sp. PCC 7120 on TiO2 Films under a Photocatalytic Device
Source: Molecules. 2023 Oct 20;28(20):7200. doi: 10.3390/molecules28207200 (PMC10609019; doi:10.3390/molecules28207200)
Supplement: Supplementary file 1 [file molecules-28-07200-s001.zip › molecules-2650806-supplementary.pdf]

In-situ observation of Cellular Structure Changes and Chain Segregations of *Anabaena sp.* PCC 7120 on TiO_2 Films under a Photocatalytic Device

Xiaoxin Wang [1,2,3,*], Jingtao Zhang [3,4], Qi Li [3,5], Ran Jia [1], Mei Qiao [1] and Wanling Cui [2]

[1] College of Physics and Electronics, Dezhou University, Dezhou 253000, China; jiaran0518@163.com (R.J.); qiaomeirr@sina.com (M.Q.)
[2] Shandong Provincial Key Laboratory of Biophysics, Dezhou University, Dezhou 253000, China; wanlingcui@163.com
[3] Shenyang National Laboratory for Materials Science, Institute of Metal Research, Chinese Academy of Sciences, Shenyang 110016, China; jtzhang@zzuli.edu.cn (J.Z.); qiliuiuc@swjtu.edu.cn (Q.L.)
[4] College of Tobacco Science and Engineering, Zhengzhou University of Light Industry, Zhengzhou 450002, China
[5] Key Laboratory of Advanced Technologies of Materials (Ministry of Education), School of Materials Science and Engineering, Southwest Jiaotong University, Chengdu 610031, China
* Correspondence: wangxx@alum.imr.ac.cn; Tel.: +86-0534-898-5834

Experimental section

Media and conditions of cell cultivation

Anabaena sp. PCC 7120 cells were grown photoautrophically on a rotary shaker at 26 ^{o}C in BG-11 medium under a 12 h : 12 h light : dark regime with white light, and harvested in the late exponential phase of growth (roughly incubated for 10 days). The culture was washed twice with the buffer solution (0.05 M KH_2PO_4 and 0.05 M K_2HPO_4, pH 7.0) by centrifugation at 6000 rps for 5 min at room temperature. The blue algae cells were resuspended in the buffer solution for the following experiments [30].

Preparation and characterization of TiO_2 film

TiO_2 thin film was prepared with a sol-gel method[19,31]. The precursor solution was prepared by first dissolving a proper amount of titanium butoxide (Ti $(OC_4H_9)_4$) in ethanol, followed with the addition of a mixture of acetic acid and distilled water dropwisely under continuous magnetic acid was 0.2 : 2.0 : 0.1 : 0.2. After being stirred for 0.5 h, a transparent and homogeneous sol solution was obtained. Prior to the sol solution deposition, glass slides were washed with detergent, distilled water, and ethanol,

respectively. Thin film samples were deposited on glass slides by spin-coating with the spinning speed of 1500 rpm, and were aged in air for 24 h to allow further hydrolysis and drying. Then, the samples were calcinated in air for at 500 ^{o}C for 1 h to obtain the desired transparent photocatalytic TiO_2 thin films. Their X-ray diffraction (XRD) analysis was conducted on a D/MAX-2004 X-ray powder diffractometer (Rigaku Corporation, Tokoyo, Japan) with Ni-filtered Cu (0.15418 nm) radiation at 56 KV and 182 mA, and their SEM observation was conducted on a SUPRA35 Field Emission Scanning Electron Microscope (ZEISS, Germany).

TiO_2 thin film characterization

Figure S1(a) showed the XRD pattern of TiO_2 thin film, which demonstrated that it had an anatase structure without rutile phase. Figure S1(b) showed the SEM image of TiO_2 thin film, which demonstrated that it had a dense surface and no microcrack was found. The crystallite size in TiO2 thin film was in the range of ~ 10 to 20 nm.

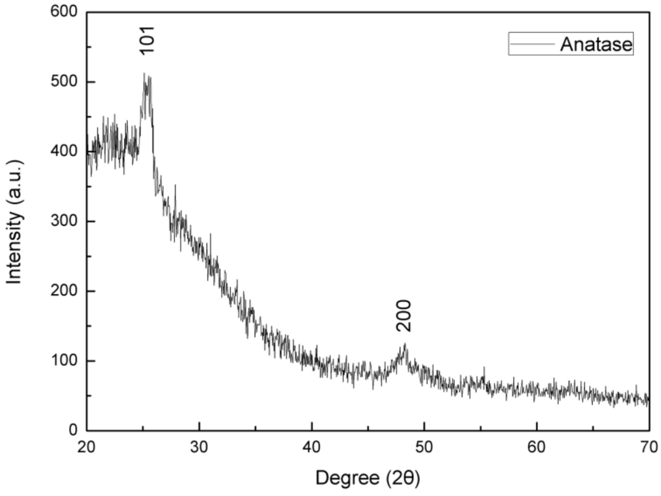

(a)

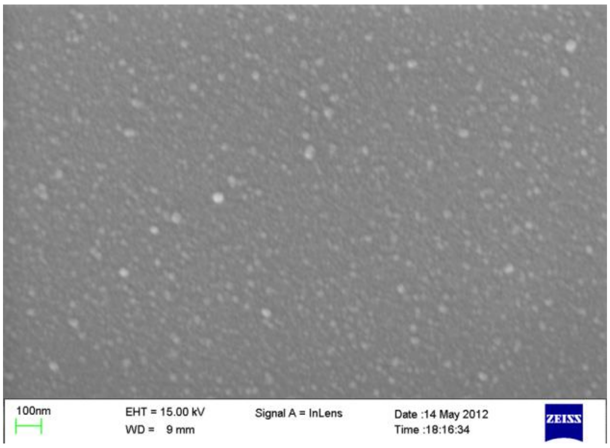

(b)

Figure S1. (a) XRD pattern and (b) SEM image of anatase TiO_2 thin film.

DAPI filter block

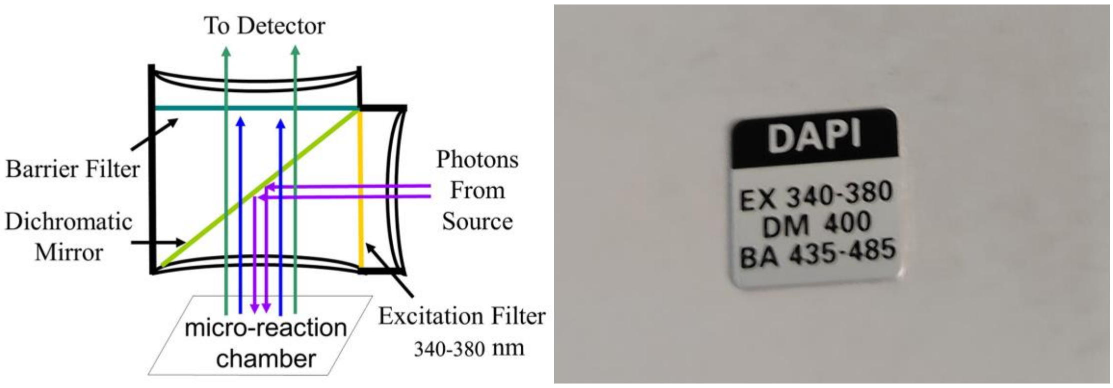

(a) (b)

Figure S2. (a) Schematic illustration of the Filter Block DAPI (Ex 340-380 nm/DM 400 nm/BA 435-485 nm) and (b) the DAPI tag along with the light source

The U-LH100HG 100 W mercury lamp was used as the light source, and the DAPI filter block was used to provide light illumination with 340 to 380 nm (see Figure S2).

The Excitation Filter (Ex 340-380 nm) only transmits light with a wavelength of 340-380 nm. The Dichromatic Mirror (DM 400 nm) transmits light with a wavelength over 400 nm, and reflects light with a wavelength below 400 nm. The Barrier Filter (BA 435-485 nm) that transmits light with a wavelength over 435-485 nm, and blocks light with a wavelength below 435-485 nm, is blocked. The Filters' information can be found on the the DAPI tag along with the light source in Figure S2(b). With the help of Excitation Filter (Ex 340-380 nm), we can eliminate the UVB influence on blue algae in the photocatalytic treatment.

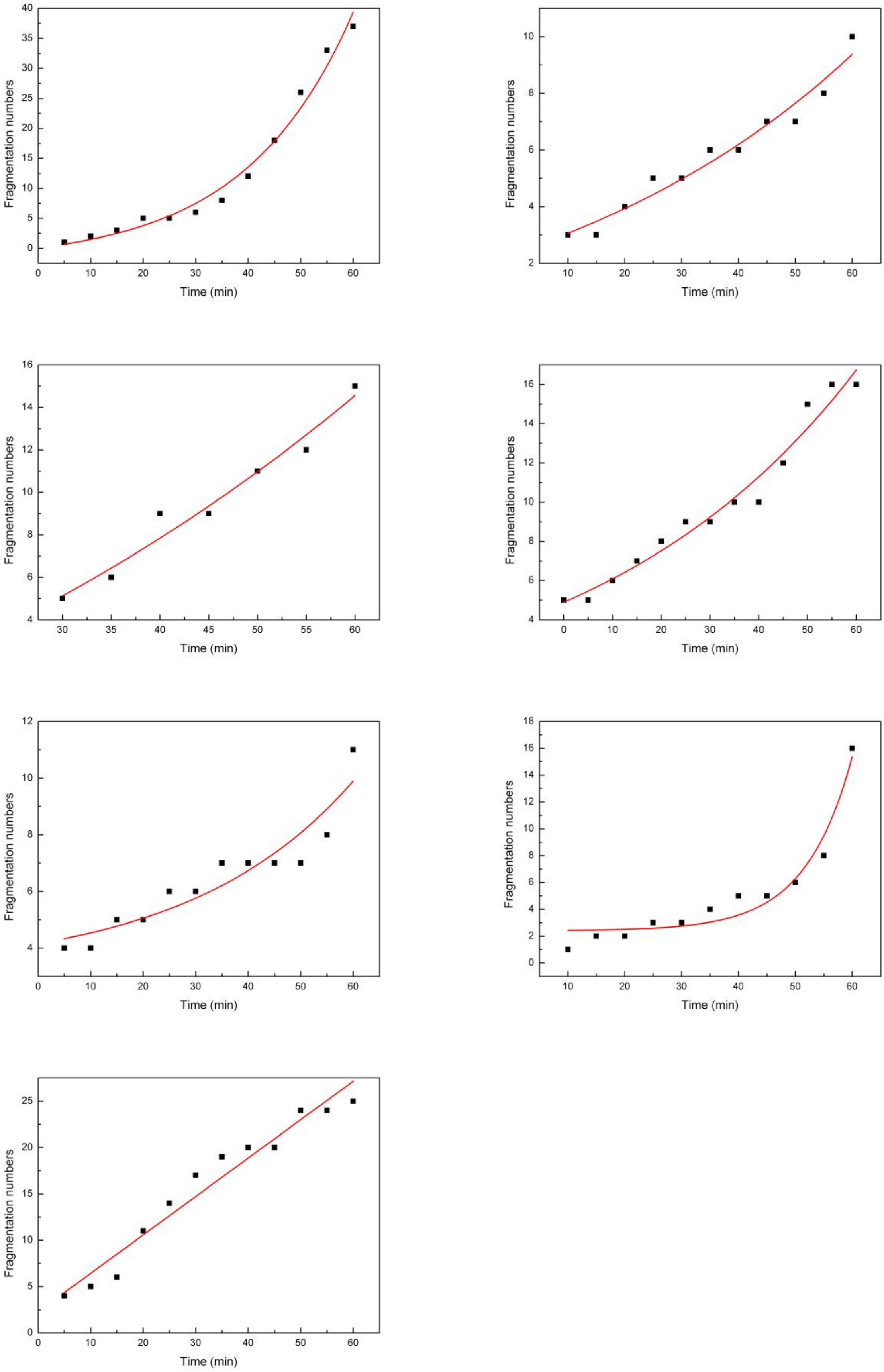

Figure S3. The relationship between chain/fragment numbers and the photocatalysis time *t* in other 7 observations.

Figure S3 demenstrated the relationship between chain/fragment numbers and the photocatalysis time t in other 7 observations, and the R^2 values for exponential fitting of these observations are 0.978, 0.950, 0.951, 0.962, 0.854, 0.938, and 0.944, respectively.

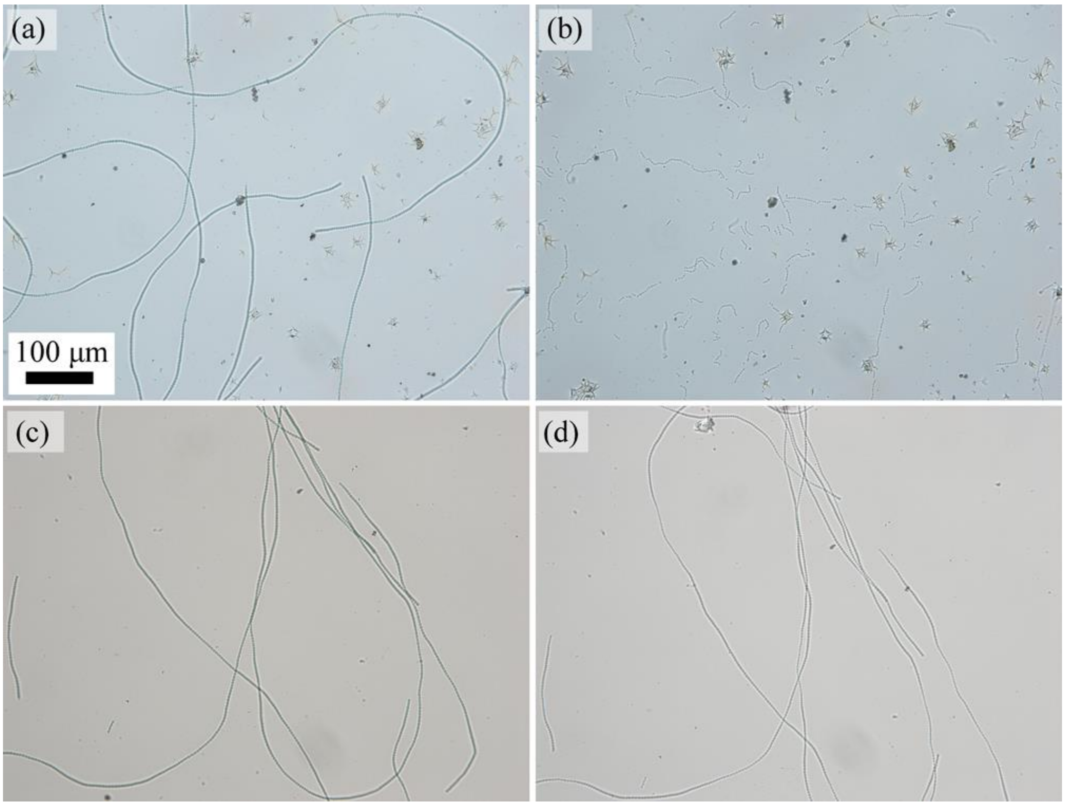

Figure S4. OM observations of *Anabaena sp.* PCC 7120
(a) treated by UVA on TiO_2 film for 0 min, (b) treated by UVA on TiO_2 film for 60 min, (c) treated by UVA for 0 min, and (d) treated by UVA for 60 min at a magnification of 200 times.

Figure S4 demonstrated the *Anabaena sp.* PCC 7120 chain structure change before and after the UVA irradiation observed in the micro-reaction chamber, compared with a control sample on a bare glass slide without TiO_2 coating. Figure S4a demonstrated that *Anabaena sp.* PCC 7120 had long, continuous, and intact chains before the UVA irradiation. The cells had a healthy green color, indicating the presence of chlorophyll a. After being photocatalytic treated by TiO_2 thin film in the micro-reaction chamber under UVA

irradiation for 60 min, lots of chain fractures happened and the original long chain structure could not be preserved. The size of these treated cells became smaller, compared with untreated ones. The original green color of these cells was dimmed, indicating the loss of chlorophyll a during the photocatalytic treatment. The observations are consistent with Figure 3 and Figure 4. When there was no TiO_2 thin film, however, no visible difference could be observed on the *Anabaena sp.* PCC 7120 chain structure before and after UV irradiation for 60 min as demonstrated in Figure S4(c) and Figure S4(d), respectively. Thus, this observation demonstrated that UVA irradiation itself could not cause huge damages on *Anabaena sp.* PCC 7120. The huge structure change demonstrated in Figure 3 and Figure 4 could only be attributed to the photocatalytic treatment by TiO_2 thin film in the micro-reaction chamber.

videoS1-The video of chain rupture process at different photocatalysis times.wmv

Video S1. The video of chain rupture process in photocatalysis.
